# Lecture 3d—Neuralgia

**Published:** 1873-01

**Authors:** Walter Hay

**Affiliations:** Chicago; Assistant to Chair of Practical Medicine, and Lecturer on Diseases of Brain and Nervous System, Rush Medical College


ARTICLE II.—LECTURE 3D—*Neuralgia.* By WALTER HAY, M.D., Assistant to Chair of Practical Medicine, and Lecturer on Diseases of Brain and Nervous System, Rush Medical College.

(Continued.)

GENTLEMEN: In my last lecture I gave you the outlines of the various forms and phases of peripheral neuralgia. I will now describe to you some of the most important forms of visceral neuralgia. Of these, that which you will most frequently meet in the course of your practice, has its location in the uterus and ovaries; indeed I may say, that these largely preponderate numerically over all other forms of this class.

This is undoubtedly due to the frequent and extreme changes which the nervous apparatus of these organs must sustain, in their passage from the highest state of physiological activity to that of complete quiescence; to the extent and complexity of this nervous mechanism; to the intimate correlation of the sexual apparatus with, not only the bodily, but with the mental functions as well; and to the remarkable susceptibility to disturbance in their functions and their anatomical condition with which these organs are endowed. Of uterine and ovarian neuralgias, one of the most common forms is that which attends menstruation, and this more especially in young subjects. It is not to be confounded with what is commonly called dysmennorrhœa, or obstructed menstruation, although the pain in this case is sometimes truly neuralgic. It will often be found to continue after the escape of the menstrual blood has been effected, and often too, indeed most frequently, when this has been quite free from the beginning, when there has been no dysmennorrhœa; indeed, some of the worst cases are those which accompany an excessive menstrual flow—mennorrhagia. The neuralgic pain seems to be independent of the flow, and to be an expression of some intrinsic quality of instability or excessive irritability in the uterine nerves or their centres. Some of these cases are indentical, pathologically, with some of the forms of trigeminal neuralgia described in my last lecture, having for their ultimate factor vaso-motor paralysis, and consequent engorgement of the ovarian vessels, and pressure upon sensory nerve fibres.

Uterine and ovarian neuralgias, (I speak of them jointly, for they are practically almost inseparable, and indistinguishable,) are not always, however, the result of direct uterine irritability, but frequently are reflex in their character, and occasioned by sources of irritation more or less remote, such as the presence of ascarides in the rectum, calculus in the bladder or in the pelvis of the kidney, tumors in the uterus or its appendages, ulceration of the neck of the uterus. It is said to have been caused by a carious tooth.

I have recently had under my own care, a case of intense uterine neuralgia, which could be attributed to no probable origin, the organ and its appendages being, and having always been, in a perfectly healthy condition.

Neuralgia of the urethra is mentioned by some authors; it is very rare. Neuralgia of the rectum sometimes occurs. In the testicle it is not uncommon. I have a patient who is subject to occasional attacks.

Occurring in the kidney or the liver, as asserted by some writers, the diagnosis is difficult—I should think, indeed, impossible. The cases reported are rare, and may, I think, be assigned to inflammation or mechanical irritation of these organs respectively.

One of the most common, most severe, and most obstinate forms of visceral neuralgia, is that which affects the heart.

The identity of cardiac neuralgia and angina pectoris is disputed by many, and asserted by others, but it appears to me that the ground for the difference of opinion is rather relative than absolute; the idea of angina pectoris having been always associated with ossified, or rather with atheromatous coronary arteries, and with essential modifications of the nutrition of the organ, seems to be totally at variance with that of cardiac neuralgia, in which there is oftentimes no *apparent* structural disease. To those who would still endeavor to preserve the fanciful distinction between functional and structural diseases, the two conditions are apparently irreconcilable. But there are many cases of angina pectoris in which structural changes have progressed to a very slight extent, and there is no case of neuralgia in which structural changes have not progressed to a very slight extent. For I will here reiterate, what I have already endeavored to impress upon you, that any modification in the functional energy of a nerve, (or of any other organ,) is necessarily preceded by change in its structural integrity.

I have just told you that many cases of angina pectoris were accompanied by no changes in the heart, recognizable by ordinary means of observation. In order to account for these changes, then, we are driven to assume a cause lying deep in the nervous system, most probably some change in the molecular organization of the nerves themselves.

The onset of cardiac neuralgia, like that of all other forms of the same disease, is sudden; the patient, without any warning, but usually after some active exertion, is seized with severe pain, resembling, in its severity and rapidity, a stab with a knife, described by most authorities as beginning at the lower part of the sternum, and extending through to the back and left shoulder, and down the left arm.

In some cases, however, and I have had frequent opportunities for observation, the pain begins, and is restricted to a small spot about an inch and a half below the nipple, corresponding to the point at which the cardiac impulse is felt against the wall of the chest. The stabbing sensation first experienced, is soon complicated with a feeling of compression, as if the heart were squeezed tightly or pinched. The countenance assumes an anxious expression, and is sometimes pale, sometimes livid; the pulse at times small and feeble, at others full and bounding, but always irregular, the prominent and constant symptom being irregularity of circulation. Under my own observation—and as I have already said, my opportunities for observing this disease have been frequent—the direct relation between the pain and the pulse has been strongly marked; the pulse stopping abruptly at the moment of the onset of the pain, during the time of one or two pulsations, and then beginning, but slowly and irregularly, recovering its rythm only after five or six beats. This relation has been so distinctly marked, that it has been easy to mark the onset and the subsidence of the pain by means of the finger applied to the pulse at the wrist, the patient's face remaining covered.

Authors describe this form of neuralgia as very rare before the age of forty. It sometimes occurs early in life; I have seen it as early as twenty-five.

In reference to the disputed identity of cardiac neuralgia, and the angina pectoris of the older writers, it may be said, that those who attribute angina to structural modifications of the heart, seem to forget that nerve tissue is as much a part of the structure of that organ as muscle or connective tissue, or any other; and that it is quite as reasonable to assume that changes in nerve tissue, such as atrophy or neuritis, may occasion disease, as that changes in muscle or connective tissue could do so. And totally unreasonable to assert that either tissue could be seriously diseased without involving the other to some extent.

There remains but one form of visceral neuralgia of sufficient importance to be specially noticed here. This is gastralgia, or gastric neuralgia, or neuralgia of the stomach, having for its seat the afferent fibres of the gastric division of the pneumogastric nerve. In this, as in all other neuralgias, the collateral evidences of debility are present. Its victims are principally women; it is in the case of these not unfrequently a reflex phenomenon, originating in uterine or ovarian disease. It is generally marked by more or less mental depression, which, like the pain, is intermittent in its character. Notwithstanding the apparent severity of the gastric disorder, the appetite is rarely interfered with. There is a peculiar feature about this disease which I believe to be pathognomonic, that is, the relief obtained by pressure; this is so apparent to the patient that he may frequently be observed beating his left side with his fist, for it is upon the left or cardiac extremity of the stomach, that the pain is most frequently felt.

Pharyngeal and laryngeal neuralgias occur most frequently in females, and as complications of hysteria. Of the former I have seen but one idiopathic uncomplicated case.

The above summary comprehends all the more prominent forms of neuralgia, which you will be liable to meet; but before leaving this portion of the subject, I wish to call your attention specially to one characteristic of the disease in general, and that is, its almost universal hereditary transmissibility. A neuralgic diathesis is rarely found without a neuralgic ancestry, or at least an ancestry marked by decided nervous instability of some sort or another. In the one generation the perturbation may involve cerebration, and in the other sensation, the insane parent generating the neuralgic offspring. Or again, in the one generation, disturbances of motor function, either in the direction of excess or defect, may be supplemented in the next by derangements of the sensory function, and in these cases we find the neuralgic offspring of epileptic or paralytic parentage. This fact of hereditary transmission underlies the whole history of nervous disease. It must not be supposed that this is restricted to the reproduction of specific forms of nervous disease, or that nervous diseases never develop in the descendants of perfectly healthy ancestry. These, like all other disturbances of health, must originate somewhere, and of course just as well in the present as in any past generation. What is transmitted from one generation to another is that peculiar instability, motility of nerve tissue, which diminishes its resistance, and renders it so liable to derangement, under the influence of determining causes which induce this or that special form of disease. Thus a patient endowed with a nervous system of this character, may under the operation of certain influences become epileptic; under others, neuralgic; under others, paralytic; under others, insane; and so on, through an immense variety of morbid conditions.

These facts, together with the numerous instances of nervous derangement arising co-incidently with neuralgia, and undoubtedly dependent on it as their exciting cause, justify us in arriving at conclusions regarding its true seat. As already referred to in my last lecture, changes in nutrition and in motor function accompanying many cases of neuralgia, indicate a remarkable uniformity in the condition of the different nerves ministering to the various functions, and which indicates that the pain of neuralgia is not necessarily dependent upon changes in the peripheral portions of the nerve, but possibly upon changes in the nervous centre itself.

If we suppose that an afferent nerve, in a state of disease, should transmit to its appropriate centre an impression, perverted by reason of its own diseased condition, the nerve-cells of the ganglion or nerve-centre, destined to receive impressions through that nerve, would not undergo their normal molecular changes, but some other, or perhaps none at all, by reason of their insusceptibility to any other than their normal impressions. But these nerve-cells in a healthy state, under the influence of their appropriate changes, induce reciprocal changes in other nerve-cells, motor or trophic, of the same nervous centre, by means of which the proper functional energy of their efferent nerve fibres is excited.

Thus, for example, if a hot body be applied to the hand or foot, the afferent or sensory nerve being in a healthy condition, transmits to the nervous centre the impression of heat. The reception of this impression necessitates a determinate and invariable change in the nerve-cells of the centre, which are designed to receive that particular impression; this change induces determinate and invariable changes in other cells of the same central ganglion, by means of which the functional energies of certain efferent nerves, (motor or trophic) are called into activity, and the appropriate changes in motion or nutrition are thus effected. The sensitive surface is withdrawn from contact with the burning body, and vesication, or some other tissue change, occurs at the surface of contact.

This embodies the whole theory of reflex action—for you observe that I excluded the influence of the will entirely.

But, on the other hand, if the afferent nerve be diseased, it cannot convey a normal impression to the centre, the appropriate cell-modifications cannot be accomplished, the motor and other energies will be excited in an abnormal manner or not at all.

Again, the negative result may occur in the case in which the afferent nerve is healthy, and entirely capable of conveying normal impressions; but the cells of the centre are unfitted by disease to sustain their appropriate changes.

Or, still further, the receptive cells in connection with the afferent nerve fibres being healthy, the motor or trophic cells associated with them in the same nervous centre, may be themselves unfitted by disease to receive and transmit their normal modifications. Either of these conditions may exist in neuralgia, and either would sufficiently account for the morbid phenomena which so frequently accompany it. But when the additional fact is stated, that in post-mortem examinations of the subject of idiopathic neuralgia, (of course those of traumatic origin are excluded,) the extremities of nerves have usually been found unchanged. From which it may be assumed that in many, if not in the majority of cases, the disease is due to the condition of the nerve centres themselves.

I have been, perhaps, a little tedious in making the above explanation; but (as I mentioned a moment since) it involves the whole theory of reflex action, and unless you thoroughly master this most important form of nervous energy, you will attempt in vain to appreciate the intricate complications of nervous pathology.

This presents a striking illustration of the conversion of force, and of the truth of the proposition that force is never lost, only converted.

Among the reflex effects of neuralgias are various affections of the skin and its appendages, developed in the manner just described. Erysipelas is occasionally observed; changes in the color of the hair are frequent. It is rare to find a neuralgic subject whose hair has not become more or less gray, in the vicinity of the affected nerves. These decolorizing changes are sometimes produced with great rapidity, it being sometimes quite possible to observe a marked increase in the grayness of the hair after a severe attack of neuralgia. It is asserted by some authors, that these changes are sometimes temporary, that the color will return to the bleached hair, upon the cessation of the pain, to be again discharged upon its recurrence. I have not observed this personally, but it is quite plausible.

Permanent changes in the nutrition of the skin have been noticed by some authorities, by which the integument assumes a glazed, shining appearance. This will be found, I think, to accompany neuralgic cases of traumatic origin, and it may be concluded, that they result rather from direct mechanical injury to trophic nerves, than to any reflex effect of neuralgia.

Thickening of bone is frequently found in old neuralgic subjects, at points near which the pain has been habitually felt; these, from being a consequence, become eventually a cause of neuralgia, by reason of the pressure which they exert upon sensory nerves. They result from the same pathological changes to which, in my last lecture, I attributed the persistent tenderness to the touch remaining after the subsidence of neuralgia attacks, *i. e.*, to a low grade of inflammatory action in the tissues contiguous to the painful nerve.

Mr. Anstie attempts to draw a distinction between reflex action, and what he considers the mode of production of some of these effects, *i. e.*, by transmission of disease from one root to another of the same nerve. This is simply shortening the circuit without changing the route. I cannot understand how an effect is to be transmitted from a root of a nerve having one function, to that of another having another and different function without the intervention of a centre; as well might he attempt to transfer a telegram to a parallel line of wire without the intervention of a registering instrument.

Modifications in the secretory action of glands may occur, and frequently do, as reflex effects of neuralgia. A prominent illustration is furnished by the increased flow of tears accompanying neuralgia of the fifth nerve; and will indicate to you how the secretions of other glands, liver, kidney and other glands, are frequently found to be so greatly perverted in the cases of old neuralgics.

In my last lecture I indicated to you the prominent characteristics of neuralgia, and this affords the bases for diagnosis. Your diagnostic marks, therefore, will be—

1. The character of the pain, in reference to duration; it is either truly and completely intermittent, with intervals of entire ease, or at least with regularly marked periods of abatement, unaccounted for by other corresponding symptoms.

2. The pain is very much more severe than the amount of general constitutional disturbance would seem to justify. In former times, when the nature of nervous diseases was little understood, many a poor sufferer has been condemned by an ignorant doctor as *nervous*, because he complained of pain, and had no fever or dirty tongue, or diarrhœa, etc., to corroborate his complaints.

3. In the vast majority of cases, the pain is unilateral, and is limited to particular nerves and their branches. When bilateral it is symmetrical.

4. Fatigue, over-exertion of any kind, but especially of the mind, aggravates the pain.

Recognizing these characteristics or diagnostic marks clearly, you will then look for evidence of—

1. A neuralgic history, either personal or hereditary.

2. For some peripheral source of irritation, such as a mechanical irritant, a wound, a scar, carious tooth, etc.; the over-exertion of some particular organ, as the eye, for example.

3. For the evidences of some blood-poison, either malarial, rheumatic, or syphilitic.

4. In old cases look for tender points, where the nerves affected emerge from the bones.

5. And lastly, for reflex effects upon the circulation, the vaso-motor nerves, upon glandular secretion, or upon nutrition of tissues, or upon motor or sensory nerves, as in local paralyses or anæsthesiæ.

Maritz Benedict describes what he terms the *curve of intensity* of pain, and applies to the detection the exact location of the disease. Thus, for example, he asserts that if the disease be located in the peripheral extremity of the nerve, the pain is continuous; if in the course of the nerve trunk, it is paroxysmal; if in the nerve roots or centres, lancinating. Anstie cites, as confirming this theory, the pains of locomotor ataxia, which are lancinating.

The theory may be correct, but one fact is not broad enough for a foundation of a theory, and it cannot be accepted without a much greater mass of evidence than this. To this authority, (Anstie), belongs the limitation of the locality of neuralgia, to atrophy of the posterior roots of the nerves. He cites much testimony to sustain his position, but the question is yet *sub judice.*

Gentlemen, if you think that I have been tedious in detailing the characteristics of neuralgia, console yourselves with the fact that anæsthesia, pain, paralysis and convulsion, are the elementary factors in the whole range of spinal diseases; comprehending these, you have a good foundation upon which to build the nervous pathology of the future.

We come now to the treatment of neuralgia; and this,while it is to the patient the most important, is to the physician the most difficult portion of the whole subject.

It would be impossible for me to give you a list of all the remedies which have from time to time been suggested for the cure of neuralgia, for time would fail me, and you have them already, in the index to Wood & Bache's Dispensatory, which just about comprises the whole of them.

The treatment, however, may be divided into prophylactic, palliative, and curative.

Under the head of prophylactics, I include diet, and regimen, and tonic.

These should all be directed to the reconstruction of broken-down nerve tissue, specially, and of tissue in general. The diet should be a generous one. There can be no greater mistake than to starve neuralgics. Too many of them starve themselves voluntarily, under the impression that their sufferings result from dyspepsia—a mistaken idea. While it is true that this latter condition is not unfrequently an accompaniment of neuralgia, it is rather a local expression of the diminished nerve-power, which has already expressed itself in the neuralgia, than a cause of the pain. For you will find, as a general rule, that increase of appetite is a pretty accurate indication of the convalescence of the patient. The essential element of neuralgia being atrophy of nerve tissue, it follows that tissue-making, histogenetic, food must be supplied for the repair of that condition.

The food should consist largely of albuminous matters—meat, eggs, and above all, milk, containing, as you know it does, oil and phosphorous nerve-food; and this you will find your patients will bear, when they can take no other food. And last, but by no means the least, fatty matters, and the best of these is cod liver oil. This is often objected to by patients, who will tell you they have tried, can't take it, can't digest it, that it occasions diarrhœa, etc., etc. These objections are all valid as far as they go, but pertain rather to the mode and manner of administration than to the substance itself. The mistake usually made in the administration of cod liver oil, is its exhibition in too large doses, usually half an ounce. Give the oil in teaspoonful doses, and not until an hour or two after eating, in this manner it is not brought into contact with the gastric surface at all, by which it cannot possibly be absorbed, but mingled with the mass of food is carried into the duodenum.

Together with nutritious food, plenty of fresh air should be administered, with as much exercise as the patient will bear short of fatigue; and, most important of all, sleep. The most powerful means of restoring wasted nerve-power, in all cases in which it has been diminished, is sleep. On no account allow the subjects of nervous disease to be deprived of sleep; for every hour taken from the natural period of rest they will have to pay a fearful penalty.

In this connection, I may advise upon one subject about which you will frequently be consulted by anxious parents, that is, upon the amount of sleep to be allowed to children. Give them, invariably, all that they need; never wake a sleeping child, nor indeed a nervous patient, when it can be avoided.

A great deal of nonsense, poetical and prosaic, has been written and spoken on the subject of early rising, but be sure to give your patient a large dose of sleep, to be taken at early bedtime. If he can sleep eight hours, he will live the longer for it, and accomplish more in his lifetime than the early riser, who will probably retire early to his grave.

Of the palliative remedies I would mention, first, local applications. These comprise hot water, aconite, chloroform, or the two combined in some emollient vehicle, such as olive oil, belladonna extract, mustard, veratria in the form of ointment, and this applied by preference to the spinal column, and perhaps the most valuable of all, blisters. I have seen sciatica which resisted every other application, yield at once to blistering. Be careful, however, that the blisters do not remain too long, so as to establish a drain upon the blood-vessels, but rather apply them in the form of flying blisters, to be removed just before vesication commences, and re-applied in the immediate vicinity.

Under the head of palliative remedies, I should include hypodermic injections of morphia, although some of the highest authorities class it among the most valuable and powerful curative agents. Some indeed place it at the head of the list. It will undoubtedly produce sleep, and thus give the irritable nervous system time to appropriate nutrition and thus to accumulate force, or rather, to speak in more accurate physiological language, to resume a condition which will enable it to convert nutritive force into nerve-force.

I think this is all the advantage which can be claimed for any of the narcotico-stimulant remedies in use. The advantage is great, I admit, and is sometimes essential to the curative treatment, but I wish you to understand distinctly the true relations of these remedies to the disease, for unless you do, you will be misled in their application, and may find that you have made more opium-eaters than you have cured neuralgics.

One of the most valuable of this class of remedies is belladonna, or preferably its alkaloid atropia, in neuralgias of the pelvic viscera, uterine or ovarian. In my own hands it has no equal. My own impression of its effect is, that it is due to its power as a vaso-motor stimulant, in inducing contraction of the pelvic arteries, cutting off the supply of blood to the irritable nerves, and diminishing pressure upon them from the engorged vessels. In hemi-crania it sometimes gives relief in the same way, I believe; to borrow from Professor Allen, "expertus loquor," that is, "I know how it is myself."

Under the head of curative treatment, I include nerve-tonics proper, that is to say, those agents which directly stimulate nerve-tissue and cause it to appropriate nerve-food from the blood, as distinguished from the effect of narcotics just referred to. These are—iron in all its forms and in large doses; the muriated tincture or tincture of the sesqui chloride; the pyrophosphate iron by hydrogen; the subcarbonate, and so on through the whole list: they are all valuable, and may be ranged about as I have mentioned them.

Strychnia is perhaps the most useful drug for the treatment of nervous diseases which we possess, while its toxic effects are manifested primarily through motor nerves. I believe its primary effect is upon the portion of the nervous system dominating the functions of nutrition. Its effect in neuralgia is sometimes most decided and rapid. It may be given alone, or, preferably, combined with iron. Phosphorus, in the form of dilute phosphoric acid, or phosphoretted oil, is highly esteemed by high authority. This agent possesses great theoretical value, in view of chemical composition of nerve-tissue into which it enters as a constituent more largely than into any other substance in nature. The objection to its use is the difficulty of administering it safely. Its compounds, phosphites and phosphates, I believe to be inert.

Prof. Hammond eulogizes highly the phosphide of zinc. I hope its effect will justify his encomiums, but I doubt it.

Arsenic, in the form of arsenite of potassa, or Fowler's solution, in five-drop doses, three times daily, largely diluted with water, and continued for a long time, five or six weeks, or even longer with intervals, is one of the most valuable nerve-tonics within our reach.

(To be continued.)

